# Investigation of the Anti-*Toxoplasma gondii* Potential of Loratadine

**DOI:** 10.3390/pathogens15070773

**Published:** 2026-07-22

**Authors:** Stephanie Ortega Alves, Ingrid de Oliveira Dias, Gabriel Candido Moura, Samuel Cota Teixeira, Juliana Quero Reimão

**Affiliations:** 1 Laboratory of Preclinical Assays and Research of Alternative Sources of Innovative Therapy for Toxoplasmosis and Other Sicknesses (Lab. PARASITTOS), Faculdade de Medicina de Jundiaí, Jundiaí 13202-550, Brazil; 2 Laboratory of Immunophysiology of Reproduction, Institute of Biomedical Science, Universidade Federal de Uberlândia, Uberlândia 38405-320, Brazil; samuel.teixeira@ufu.br

**Keywords:** *Toxoplasma gondii*, toxoplasmosis, drug repurposing, loratadine, antihistamines, *in vivo* efficacy

## Abstract

Toxoplasmosis remains a major global health concern, particularly in immunocompromised individuals and pregnant women, while currently available therapies are associated with significant toxicity and limited efficacy against chronic infection. Drug repurposing represents an attractive strategy for the identification of safer and more effective anti-*Toxoplasma gondii* agents. This study investigated the antiparasitic potential of loratadine, a second-generation antihistamine, using an integrated *in silico*, *in vitro*, and *in vivo* approach. The anti-*T. gondii* activity of loratadine was evaluated through β-galactosidase-based proliferation assays, reversibility assays, and experiments using pretreated tachyzoites. *In vivo* efficacy was assessed in murine models of acute and chronic toxoplasmosis. Disease progression, survival, parasite burden, and cerebral cyst formation were analyzed following treatment. Loratadine inhibited the intracellular proliferation of *T. gondii* tachyzoites in a concentration-dependent manner and induced partially irreversible effects on parasite viability after drug withdrawal. Pretreatment of extracellular tachyzoites produced limited effects, suggesting that loratadine predominantly acts during the intracellular stage of infection. In the acute toxoplasmosis model, loratadine treatment delayed disease progression, prolonged animal survival, and significantly reduced the number of tachyzoites recovered from the peritoneal cavity. In the chronic infection model, treatment significantly decreased both the number and size of cerebral cysts, although these findings do not demonstrate cyst eradication or direct bradyzoite killing. Despite its measurable biological activity, loratadine exhibited a relatively low *in vitro* selectivity index, highlighting the need for further optimization. These exploratory findings identify loratadine as a promising lead compound (hit) for further optimization rather than an immediately translatable therapeutic candidate. Additional medicinal chemistry, pharmacokinetic, mechanistic, and confirmatory preclinical studies will be required to determine its potential for anti-*T. gondii* drug development.

## 1. Introduction

Toxoplasmosis is a globally distributed parasitic disease caused by *Toxoplasma gondii*, an obligatory intracellular protozoan that infects approximately one-third of the world’s population [1]. Although infection is often asymptomatic in immunocompetent individuals, it can cause severe and even life-threatening manifestations in immunocompromised patients and congenitally infected fetuses. Reactivation of latent infection in individuals with AIDS or undergoing immunosuppressive therapy can result in toxoplasmic encephalitis, a condition associated with high morbidity and mortality. In pregnant women, primary infection may lead to vertical transmission, resulting in miscarriage, hydrocephalus, chorioretinitis, or other neurological and ocular sequelae in the fetus [2].

Current treatment regimens for toxoplasmosis are based on the combination of pyrimethamine and sulfadiazine, often administered with folinic acid to reduce hematological toxicity [3]. Despite their efficacy against the tachyzoite stage, these drugs fail to eliminate the latent bradyzoite cysts in tissues, particularly in the brain and muscle. Moreover, adverse effects such as bone marrow suppression, hypersensitivity reactions, and teratogenicity limit their use, especially in pregnant women. These drawbacks highlight the urgent need for safer and more effective therapeutic alternatives, capable of treating both acute and chronic stages of the infection [4].

Drug discovery efforts for toxoplasmosis have been hindered by the lack of compounds able to cross the blood–brain barrier (BBB) and act on encysted parasites [5]. In this context, drug repurposing—the strategy of identifying new therapeutic uses for approved drugs—has emerged as an attractive approach. This strategy reduces the time, cost, and risk associated with drug development, since repurposed compounds already have known pharmacokinetic and safety profiles [6]. In addition, repurposing enables the systematic evaluation of approved drugs with previously unrecognized antiparasitic properties, providing an efficient strategy to identify novel chemical starting points for anti-*T. gondii* drug discovery. An ideal drug for toxoplasmosis should therefore combine high antiparasitic efficacy with low toxicity, be safe for use during pregnancy, and reach effective concentrations in the central nervous system to eradicate tissue cysts and prevent reactivation episodes [7].

Among the drug classes explored for repurposing, antihistamines have gained attention for their unexpected antiparasitic properties. Beyond their well-known role in modulating allergic responses through histamine H1 receptor antagonism [8], several antihistamines have demonstrated inhibitory activity against human parasites such as *Leishmania* [9], *Trypanosoma* [10], and *Schistosoma mansoni* [11]. Although these parasites are taxonomically and biologically distinct from *T. gondii*, these findings suggest that antihistamines may exert antiparasitic effects across diverse parasite groups through different parasite- or host-directed mechanisms. However, no validated molecular target has yet been identified for loratadine in apicomplexan parasites, and whether its antiparasitic activity results from direct effects on the parasite, modulation of host–cell pathways, or other mechanisms remains unknown. These findings suggest that antihistamines represent an attractive class of compounds for exploratory drug repurposing studies against toxoplasmosis.

Loratadine is a second-generation H1 antihistamine widely used for the treatment of allergic rhinitis and urticaria [12]. It exhibits favorable pharmacokinetic properties, including high oral bioavailability and a relatively long plasma half-life, as well as a safety profile that allows its use in pregnant women [13]. Although considered a peripherally acting antihistamine, loratadine and its active metabolite, desloratadine, can cross the BBB under certain physiological and pathological conditions [14]. This feature, together with its established clinical safety and previously reported broad-spectrum antiparasitic activity, makes loratadine a compelling candidate for proof-of-concept evaluation as a repurposed agent against *T. gondii*.

Based on these considerations, this study aimed to investigate the effects of loratadine as a potential repositioned agent for toxoplasmosis. Rather than assuming a defined mechanism of action, we sought to determine whether the previously reported antiparasitic activity of loratadine could be extended to *T. gondii* through an integrated *in silico*, *in vitro*, and *in vivo* evaluation. The work focused on evaluating its anti-*T. gondii in vitro* activity, and its efficacy in experimental models of toxoplasmosis, including acute and chronic murine infection, thereby providing proof-of-concept evidence to support future mechanistic studies and medicinal chemistry optimization.

## 2. Materials and Methods

### 2.1. Drugs and Reagents

Loratadine and pyrimethamine (PYR) were obtained from Sigma-Aldrich (St. Louis, MO, USA). For *in vitro* experiments, compounds were dissolved in dimethyl sulfoxide (DMSO) to prepare 10 mM stock solutions and subsequently diluted in culture medium immediately before use. The final concentration of DMSO in all assays did not exceed 0.2% (*v*/*v*). For *in vivo* studies, commercially available loratadine tablets (10 mg; CIMED^®^, Avenida Angélica, Brazil) were purchased from a local pharmacy and processed as described below for oral administration.

### 2.2. In Silico Pharmacokinetics and Toxicity Predictions

The pharmacokinetic profile of loratadine was predicted using the SwissADME web server (http://www.swissadme.ch, accessed on 2 June 2025), which provides estimates of absorption, distribution, metabolism, and excretion (ADME) parameters. Gastrointestinal absorption and blood–brain barrier (BBB) permeability were specifically evaluated to infer oral bioavailability and central nervous system exposure [15]. *In silico* toxicity predictions, including mutagenicity, tumorigenicity, reproductive, and irritant potential, were assessed using the OSIRIS Property Explorer version 2 [16].

### 2.3. Cell Lines and Parasite Culture

Human foreskin fibroblasts (HFF) (ATCC, SCRC-1041) were maintained as confluent monolayers in Dulbecco’s Modified Eagle Medium (DMEM; Thermo Fisher Scientific, Waltham, MA, USA) supplemented with 10% fetal bovine serum (FBS), 2 mM L-glutamine, and 20.9 µM gentamicin (D10 medium) [17].

*Toxoplasma gondii* tachyzoites of the RH strain expressing a transgenic copy of β-galactosidase (Type I, clone 2F1) [18] were continuously propagated in confluent HFF monolayers. Parasites were maintained in DMEM supplemented with 2% FBS, 2 mM L-glutamine, and 20.9 µM gentamicin (D2 medium). All cell and parasite cultures were incubated at 37 °C in a humidified atmosphere containing 5% CO_2_ [17].

### 2.4. In Vitro Assays to Evaluate the Anti-T. gondii Activity of Loratadine

The *in vitro* activity of loratadine against *T. gondii* tachyzoites was assessed using proliferation reversibility and infectivity assays, as previously described [19], with minor adaptations.

#### 2.4.1. *T. gondii* Proliferation Assay

Human foreskin fibroblasts (HFFs) were seeded in 96-well plates at a density of 1 × 10^4^ cells per well in D10 medium and incubated for 18 h at 37 °C in a humidified atmosphere containing 5% CO_2_ to allow cell adhesion. Subsequently, the monolayers were infected with *T. gondii* tachyzoites at a multiplicity of infection (MOI) of 1:1 in D2 medium and incubated for 3 h under the same conditions. After infection, the medium was removed, and wells were washed with phosphate-buffered saline (PBS) to eliminate non-internalized parasites. Cells were then treated with loratadine at the indicated concentrations and incubated for 72 h. This endpoint was selected because it corresponds to the standardized β-galactosidase assay previously validated for the RH-2F1 reporter strain, allowing reliable quantification of parasite proliferation under the experimental conditions used (MOI 1:1). Furthermore, the longer incubation period increases the dynamic range of the assay and facilitates the detection of compounds exhibiting delayed antiparasitic activity without compromising host cell monolayer integrity. Parasite proliferation was quantified by measuring β-galactosidase activity.

#### 2.4.2. Reversibility Assay

To determine whether the antiparasitic effect of loratadine is reversible, HFF were infected with tachyzoites (MOI 1:1) for 3 h, followed by removal of non-internalized parasites through PBS washing. Infected cultures were then treated with loratadine for 24 h. After this period, the drug-containing medium was replaced with fresh, drug-free medium, and cells were incubated for an additional 48 h. Parasite viability was subsequently assessed by β-galactosidase activity.

#### 2.4.3. Intracellular Proliferation Assays Using Pretreated Tachyzoites—Infectivity Assay

To investigate whether loratadine directly affects parasite infectivity and intracellular development, tachyzoites were pretreated prior to host cell infection. Briefly, parasites were incubated with loratadine for 1 h at 37 °C, followed by centrifugation (1900× *g* for 7 min) and washing with PBS to remove residual compound. HFF cells, previously seeded as described above, were then infected with pretreated tachyzoites at an MOI of 1:1. After 3 h of infection, extracellular parasites were removed by washing with PBS. In the first experimental condition, β-galactosidase activity was measured immediately after this step to assess parasite invasion. In the second condition, infected cells were further incubated for 72 h prior to β-galactosidase quantification to evaluate intracellular proliferation.

#### 2.4.4. β-Galactosidase Assay and Data Analysis

Parasite viability in all assays was quantified based on β-galactosidase activity, as previously described [20]. Briefly, 50 µL of PBS containing 0.6% Triton X-100 for cell lysis and 500 µmol/L chlorophenol red-β-D-galactopyranoside (CPRG) as substrate were added to each well [21]. After observing a change in color, absorbance was measured at 570 nm using a multimode microplate reader (Varioskan Lux, Thermo Fisher Scientific, Waltham, MA, USA).

Pyrimethamine was used as a positive control, while parasites exposed to the highest concentration of DMSO used to solubilize loratadine served as the negative control. For each independent experiment, β-galactosidase activity was first normalized to the mean absorbance of the corresponding DMSO-treated control group, which was defined as 100% parasite survival. The normalized values obtained from independent experiments were subsequently pooled for statistical analysis and graphical presentation. Data was normalized and expressed as the percentage of parasite survival relative to untreated controls. All experiments were performed in at least three independent assays, each conducted with three technical replicates per condition.

### 2.5. Determination of Half-Maximal Cytotoxic Concentration (CC_50_) in Human Cells

HFF cells were seeded into 96-well plates at a density of 5 × 10^3^ cells per well and incubated overnight. Cells were then exposed to serial dilutions of loratadine (1.56–200 µM) prepared in D10 medium and incubated for 72 h. Cell viability was assessed using the resazurin reduction assay [22]. Briefly, resazurin (0.15 mg/mL) prepared in sterile PBS was added to each well (10 µL per well), followed by incubation for 4 h at 37 °C. Fluorescence was measured at excitation/emission wavelengths of 565/590 nm using a microplate fluorimeter (Varioskan Lux, Thermo Fisher Scientific, Waltham, MA, USA). Pyrimethamine was included as a reference compound, and cells incubated with the highest volume of DMSO were used as negative controls. The selectivity index (SI) was calculated as the ratio between CC_50_ and EC_50_ values.

### 2.6. In Vivo Efficacy Studies

Swiss mice were selected due to their well-established susceptibility to *T. gondii* infection and widespread use in experimental toxoplasmosis models. Female mice were used in the acute infection model due to their higher susceptibility to infection and more pronounced clinical manifestations, which facilitate the evaluation of treatment effects on disease progression and survival. In contrast, male mice were used in the chronic model to reduce variability in long-term infection outcomes and to ensure stable establishment of brain cysts, as sex-dependent differences in susceptibility to *T. gondii* infection have been previously reported [23].

Sample sizes were defined based on previous studies using similar murine models of toxoplasmosis and were sufficient to detect biologically relevant differences in survival and parasite burden. Due to ethical considerations and the use of well-established infection models with high reproducibility, group sizes of 4–5 animals were considered appropriate for exploratory preclinical evaluation. Statistical significance was assessed using non-parametric tests suitable for small sample sizes.

Animals were randomly allocated to treatment groups after infection. Parasite burden quantification and brain cyst area measurements were performed by investigators blinded to the treatment groups whenever possible to minimize observer bias.

Mice (4–5 weeks old, weighing 15 to 25 grams) used in both acute and chronic models of toxoplasmosis were obtained from the Centro Multidisciplinar para Investigação Biológica na Área da Ciência em Animais de Laboratório (CEMIB) of Universidade Estadual de Campinas (UNICAMP), Brazil.

#### 2.6.1. Acute Toxoplasmosis Model: Survival Monitoring

Female Swiss mice were infected intraperitoneally with 2.5 × 10^5^ *T. gondii* RH strain tachyzoites suspended in 100 µL of sterile 0.9% saline [17]. After infection, animals were randomly assigned to experimental groups (n = 5 per group). The untreated group received vehicle alone (5% DMSO, 5% PEG, 5% Tween 80 in 0.8% saline), whereas the treated group received loratadine at 2 mg/kg/day by oral gavage. Treatment was initiated 24 h post-infection and continued for 10 consecutive days. The same loratadine formulation (2 mg/kg/day) and corresponding vehicle were administered throughout the entire treatment period. Loratadine was administered in a final volume of 100 µL per animal. Control animals received the same vehicle under identical conditions. Animals were monitored daily for clinical signs and mortality throughout the experiment.

#### 2.6.2. Acute Toxoplasmosis Model: Parasite Load Quantification

Male Swiss mice (4 weeks old) were infected intraperitoneally with 2 × 10^3^ *T. gondii* RH strain tachyzoites suspended in 100 µL of sterile 0.9% saline [24]. After infection, animals were randomly assigned to experimental groups (n = 5 per group). The untreated group received vehicle alone (5% DMSO, 5% PEG, 5% Tween 80 in 0.8% saline), whereas the treated group received loratadine at 2 mg/kg/day by oral gavage. Treatment was initiated 24 h post-infection and continued for 9 consecutive days. The same loratadine formulation (2 mg/kg/day) and corresponding vehicle were administered throughout the entire treatment period. Loratadine was administered in a final volume of 100 µL per animal. Control animals received the same vehicle under identical conditions. At the 10th day post-infection, euthanasia was performed on all remaining mice, and the peritoneal parasite load was estimated using a Neubauer chamber. Parasites were recovered from the peritoneal cavity by lavage with sterile saline, followed by immediate counting using a Neubauer hemocytometer under light microscopy.

#### 2.6.3. Chronic Toxoplasmosis Model: Parasite Quantification

Brain cysts of the *T. gondii* ME49 strain were obtained from chronically infected donor mice and maintained by serial passage *in vivo*. Cysts were isolated from brain homogenates in sterile saline and quantified by light microscopy prior to infection. For experimental infection, male mice were orally inoculated with 10 cysts suspended in 100 µL of sterile saline [17,24]. Animals were randomly distributed into experimental groups (n = 4 per group). The untreated group received vehicle alone (5% DMSO, 5% PEG, 5% Tween 80 in 0.8% saline), while the treated group received loratadine at 2 mg/kg/day by oral gavage. Treatment began at 41 days post-infection, corresponding to the chronic phase of toxoplasmosis, and was maintained for 10 consecutive days, with a final administration volume of 200 µL per animal. The higher administration volume used in the chronic model (200 µL) compared to the acute model (100 µL) reflects the increased body weight of older animals at this stage of infection, ensuring consistent dosing (mg/kg) across experimental groups.

At day 51 post-infection, animals were euthanized by cervical dislocation after anesthesia using Ketamine (100 mg/kg) and Xylazine (10 mg/kg), and brains were aseptically removed and mechanically homogenized in sterile saline. Each brain was homogenized in a defined volume of sterile saline using gentle mechanical disruption to ensure uniform cyst recovery. The homogenate was thoroughly mixed, and aliquots were examined under bright-field microscopy for cyst quantification. Total cyst burden was determined by direct microscopic counting of all cysts observed in the analyzed aliquots and expressed as the estimated total number of cysts per brain. For cyst area determination, digital images of individual cysts were acquired using a light microscope equipped with a digital camera. Cyst area (µm^2^) was determined using ImageJ software version 1.54 (National Institutes of Health, Bethesda, MD, USA) following calibration with a stage micrometer. All image analyses were performed using identical acquisition settings and measurement parameters.

### 2.7. Statistical Analysis

Dose–response curves obtained in the *in vitro* proliferation and cytotoxicity assays were generated by nonlinear regression using a sigmoidal dose–response model with variable slope to calculate EC_50_ and CC_50_ values. For comparisons among multiple treatment groups in the *in vitro* assays, data were analyzed by one-way analysis of variance (ANOVA) followed by Dunnett’s multiple comparisons test using the DMSO-treated group as the reference control. Data related to parasite burden in mice groups were analyzed using the non-parametric Mann–Whitney test. The log-rank (Mantel–Cox) test was used in survival analysis. Statistical analyses were performed with GraphPad Prism 9. Differences were considered statistically significant when p < 0.05.

## 3. Results

### 3.1. In Silico ADMET Profile of Loratadine

*In silico* pharmacokinetic and toxicity analyses revealed a favorable ADMET profile for loratadine when compared with drugs currently used in the treatment of toxoplasmosis (Table 1). Loratadine was predicted to exhibit high gastrointestinal absorption and to be permeable to the blood–brain barrier (BBB), two properties considered critical for effective systemic and central nervous system activity against *T. gondii*. In addition, no risks of mutagenicity, tumorigenicity, reproductive toxicity, or irritancy were predicted for loratadine.

To contextualize these findings, pyrimethamine, sulfadiazine, and spiramycin were included in the comparative analysis. While pyrimethamine was also predicted to cross the BBB, it displayed unfavorable toxicity predictions, including mutagenic, tumorigenic, and reproductive risks. In contrast, sulfadiazine and spiramycin showed safer toxicity profiles but were not predicted to penetrate the BBB. Moreover, spiramycin was associated with low predicted gastrointestinal absorption and an increased risk of irritant effects.

Overall, loratadine combined high predicted oral bioavailability and BBB permeability with a favorable toxicity profile, distinguishing it from the reference drugs commonly used in toxoplasmosis therapy. These results support the selection of loratadine for further experimental evaluation in *T. gondii* infection models.

### 3.2. Loratadine Exhibits In Vitro Activity Against T. gondii

**Figure 1:**
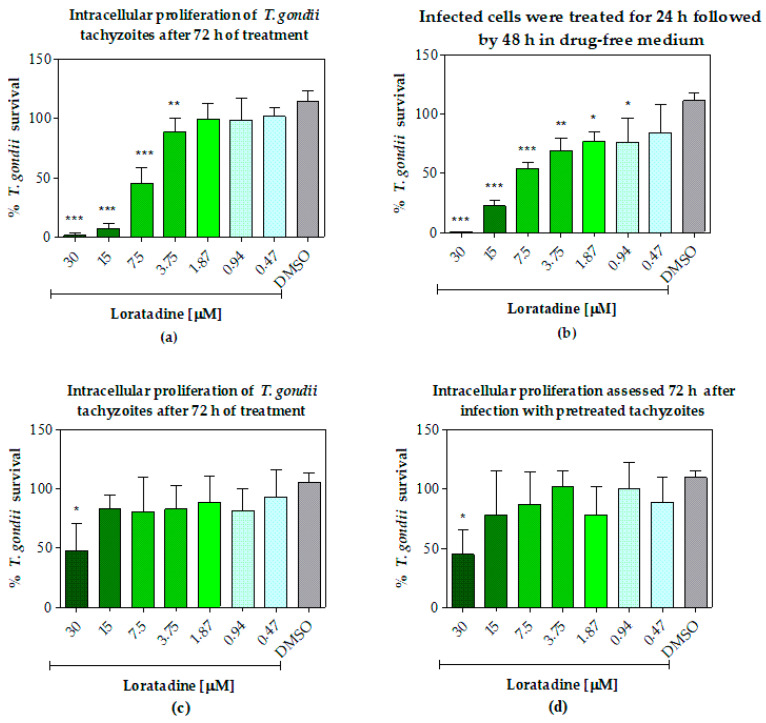
Effect of loratadine on *Toxoplasma gondii* proliferation, reversibility, and infectivity. Bar graphs showing the percentage of *T. gondii* survival after treatment with loratadine at concentrations ranging from 30 to 0.47 µM, compared with the DMSO-treated control group. Data are presented as mean ± standard deviation (SD) from three independent experiments performed in duplicate. (**a**) Intracellular proliferation of *T. gondii* tachyzoites after 72 h of treatment. (**b**) Reversibility assay, in which infected cells were treated for 24 h followed by 48 h in drug-free medium. (**c**) Parasite invasion assessed 3 h after infection using tachyzoites pretreated with loratadine for 1 h. (**d**) Intracellular proliferation assessed 72 h after infection with pretreated tachyzoites. Statistical significance was determined in comparison with the DMSO control group: *** p < 0.001; ** p < 0.01; * p < 0.05.

Loratadine exhibited a concentration-dependent inhibitory effect on *T. gondii* proliferation *in vitro* (Figure 1a). Treatment with 30, 15, and 7.5 µM significantly reduced parasite proliferation compared with the DMSO-treated control group (p < 0.001), while a moderate but still significant reduction was observed at 3.75 µM (p < 0.01). Lower concentrations did not produce statistically significant effects, indicating a threshold for antiparasitic activity under the experimental conditions.

The reversibility assay further demonstrated that the antiparasitic effect of loratadine was not reversible (Figure 1b). Significant reductions in parasite viability were observed at 30, 15, and 7.5 µM (p < 0.001), as well as at 3.75 µM (p < 0.01). Notably, even lower concentrations (1.87 and 0.94 µM) resulted in modest but statistically significant inhibition compared with the DMSO control (p < 0.05), suggesting a sustained effect on parasite viability after drug removal.

In contrast, pre-exposure of tachyzoites to loratadine for 1 h prior to infection had a limited impact on parasite infectivity and intracellular proliferation (Figure 1c,d). A statistically significant reduction was observed only at the highest concentration tested (30 µM) when compared with the DMSO control (p < 0.05), both immediately after invasion (3 h; Figure 1c) and after 72 h of intracellular replication (Figure 1d). No significant differences were detected at lower concentrations, indicating that short-term exposure of extracellular parasites to loratadine is insufficient to markedly impair their infectivity or subsequent replication.

The compound presented a half-maximal effective concentration (EC_50_) of 7.0 ± 0.9 µM in infected human foreskin fibroblasts (HFF) in the proliferation assay. Evaluation of host cell cytotoxicity revealed a half-maximal cytotoxic concentration (CC_50_) of 15.5 ± 4.5 µM, resulting in a selectivity index (SI) of 2.2 for HFF (Table 2).

Pyrimethamine, included as a reference drug, displayed a markedly higher antiparasitic potency, with an EC_50_ of 0.2 ± 0.1 µM and a CC_50_ > 50 µM, corresponding to a SI > 250.

### 3.3. Loratadine Displays In Vivo Efficacy in Murine Models of Acute and Chronic Toxoplasmosis

**Figure 2:**
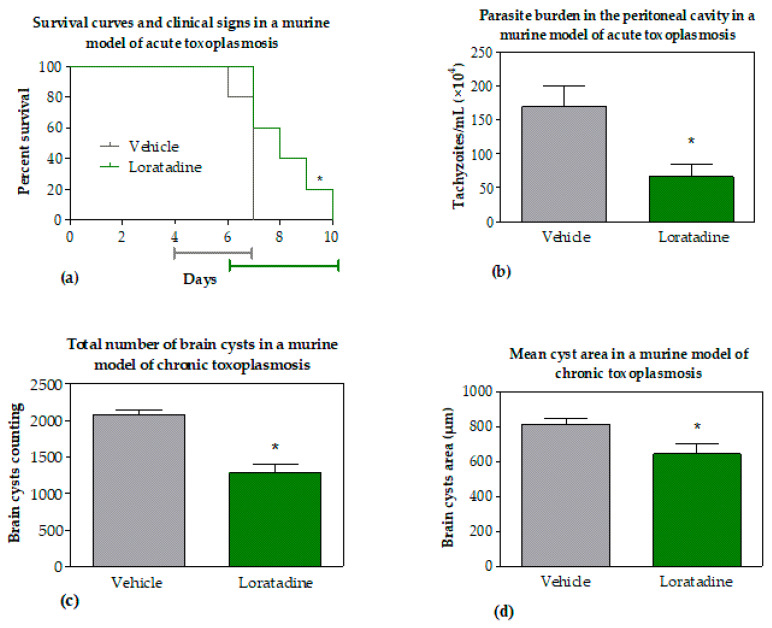
Effects of loratadine in murine models of acute and chronic toxoplasmosis. Female Swiss mice intraperitoneally infected with *Toxoplasma gondii* RH strain tachyzoites were treated with loratadine (2 mg/kg/day, oral gavage) or vehicle starting 24 h post-infection. (**a**) Kaplan–Meier survival curves and clinical signs in mice infected with 2.5 × 10^5^ tachyzoites (n = 5/group) and treated for 10 consecutive days. Statistical significance was determined using the log-rank (Mantel–Cox) test. (**b**) Parasite burden in the peritoneal cavity of male mice infected with 2 × 10^3^ tachyzoites and treated for 9 consecutive days (loratadine, n = 5; vehicle, n = 4). Tachyzoites were quantified by microscopic counting using a Neubauer chamber and expressed as tachyzoites/mL. Statistical significance was determined using the Mann–Whitney test. Male Swiss mice orally infected with *T. gondii* ME49 strain cysts were treated with loratadine (2 mg/kg/day) or vehicle for 10 consecutive days beginning at 41 days post-infection. (**c**) Total number of brain cysts. (**d**) Mean cyst area determined by morphometric analysis using ImageJ software. Data are presented as mean ± SD. Differences were considered statistically significant at p < 0.05 compared with the vehicle-treated group. The asterisk (*) indicates a significant difference compared to the control group.

To assess the impact of loratadine on disease progression during acute toxoplasmosis, mice were infected with a high parasite burden (2.5 × 10^5^ tachyzoites) of a virulent strain (RH) and monitored daily for clinical signs and survival. Untreated animals rapidly developed severe clinical manifestations, including lethargy, ruffled fur, and impaired mobility, and succumbed to infection by approximately day 6 post-infection. In contrast, mice treated with loratadine (2 mg/kg/day) exhibited a delayed onset of clinical signs and a slower progression of disease severity, as indicated by the bars below the graph (Figure 2).

Survival analysis demonstrated that loratadine treatment significantly prolonged mouse survival compared with the untreated control group (p < 0.05). While all animals in the control group died by day 6 post-infection, a subset of loratadine-treated mice remained alive until day 10 post-infection, indicating a partial protective effect in this acute infection model (Figure 2a).

To further investigate the effect of loratadine on parasite replication under conditions that allow extended survival, an additional acute toxoplasmosis experiment was performed using a lower inoculum (2 × 10^3^ tachyzoites per animal). This approach enabled the assessment of peritoneal parasite burden prior to widespread mortality. During the experimental period, only one death was observed in the vehicle-treated control group by day 9 post-infection, which defined the endpoint of the assay.

Quantification of tachyzoites recovered from the peritoneal cavity revealed a significant reduction in parasite load in mice treated with loratadine (2 mg/kg/day) compared with the vehicle-treated group (p < 0.05) (Figure 2b). Loratadine treatment reduced the number of peritoneal tachyzoites by 60.7% compared to the vehicle-treated group. These findings further support the antiparasitic effect of loratadine *in vivo*, demonstrating its ability to limit parasite proliferation under conditions of reduced infection pressure and prolonged host survival.

In a murine model of chronic toxoplasmosis, male Swiss mice infected with the *T. gondii* ME49 strain were treated with loratadine beginning at 41 days post-infection, a time point corresponding to established chronic infection. Compared with vehicle-treated controls, loratadine administration resulted in a significant reduction in cerebral parasite burden. As shown in Figure 2c, mice receiving loratadine exhibited a marked decrease in the total number of brain cysts, indicating an effect of the drug on parasite persistence within the central nervous system.

In addition to the quantitative reduction in cyst burden, morphometric analysis revealed a significant decrease in the mean cyst area in loratadine-treated animals relative to controls (Figure 2d). A reduction of 38.3% in brain cyst burden was observed in loratadine-treated animals. This finding suggests that loratadine affected not only the number but also the mean area of *T. gondii* cysts in the brain.

## 4. Discussion

This study evaluated loratadine as a lead compound for future optimization against *T. gondii* using a combined *in silico*, *in vitro* and *in vivo* pipeline. Three main findings emerge: (i) *in silico* ADMET profiling predicted high oral absorption, BBB permeability and a favorable toxicity profile for loratadine; (ii) loratadine inhibited *T. gondii* intracellular growth *in vitro* but displayed relatively low selectivity in HFF; and (iii) exploratory murine studies demonstrated measurable biological effect in both acute and chronic infection, including delayed clinical deterioration, prolonged survival in the acute model, and reductions in both the number and mean area of brain cysts in chronically infected mice. Taken together, these findings provide proof-of-concept evidence supporting further investigation of loratadine as a lead compound for anti-*T. gondii* drug discovery rather than as a clinically translatable therapeutic candidate.

The favorable *in silico* profile we observed (high predicted GI absorption, BBB permeation and absence of predicted mutagenicity/tumorigenicity) should be interpreted as complementary to the extensive experimental pharmacological information already available for loratadine rather than as novel evidence of its pharmacokinetic properties. The ADMET analysis was included to provide a standardized comparison with other repurposing candidates evaluated using the same computational workflow. Importantly, the predicted oral absorption and BBB permeability are consistent with published pharmacokinetic studies demonstrating good oral bioavailability and systemic exposure following oral administration of loratadine [25]. Drug repurposing screens and reviews underscore the strategic value of prioritizing compounds with known human safety and CNS availability when addressing chronic brain infections [25]. Such an approach has been fruitful for identifying other candidate molecules against *T. gondii* [22].

Antihistamines have previously been reported to exert antiparasitic effects across a range of organisms, including *S. mansoni* [11] and *Leishmania* spp. [9], and several reviews emphasize the potential of this class for antimicrobial repurposing [26]. Notably, second-generation antihistamines differ in BBB penetration and pharmacokinetic behavior, which influences translational potential for CNS infections; loratadine has been reported to reach the CNS in animal studies [27], and is predicted to be BBB-permeant *in silico*, supporting the observed reduction in brain cyst burden. However, despite these encouraging findings, no validated molecular target has yet been identified for loratadine in *T. gondii* or other apicomplexan parasites. Consequently, the molecular basis underlying its antiparasitic activity remains unknown and warrants dedicated mechanistic investigations aimed at determining whether the observed effects result from direct parasite inhibition, modulation of host–cell pathways, or other biological mechanisms.

Loratadine demonstrates a measurable *in vitro* antiparasitic effect against *T. gondii*; however, its relatively low SI compared to pyrimethamine represents one of the principal limitations of the present study, indicating a narrow separation between antiparasitic activity and host–cell toxicity under the experimental conditions employed. Accordingly, the present data do not support considering loratadine as an immediately translatable therapeutic option for toxoplasmosis. Rather, the compound should be viewed as an exploratory hit that provides proof-of-concept for further medicinal chemistry optimization. Structural modification aimed at improving potency while reducing host–cell toxicity will likely be necessary before clinical translation can be considered. Nevertheless, it is important to recognize that *in vitro* selectivity does not invariably predict *in vivo* efficacy, particularly for compounds that may exert pleiotropic or host-directed effects. These findings are consistent with the broader drug repurposing literature, in which initial hits often require further optimization before progressing through the drug development pipeline [28]. In this context, the evaluation of other clinically approved antihistamines may provide valuable insights into class-related antiparasitic activity and help identify compounds with improved selectivity profiles. Such comparative analyses could contribute to the identification of more potent and selective candidates, supporting the repositioning of antihistamines as a promising strategy for anti-*T. gondii* drug discovery.

An apparent discrepancy exists between the modest *in vitro* potency of loratadine and its measurable efficacy in the murine models. Notably, pharmacokinetic studies in mice previously reported demonstrated that oral administration of loratadine (20 mg/kg) produced peak plasma concentrations of approximately 133 ng/mL (≈0.35 μM), substantially lower than the EC_50_ determined in the present *in vitro* assays [29]. Although direct comparisons between studies should be made cautiously due to differences in dosing regimens and experimental conditions, these observations suggest that the *in vivo* activity observed here may not be fully explained by the direct action of the parent compound alone. Alternative, non-mutually exclusive hypotheses include the contribution of active metabolites such as desloratadine, preferential tissue distribution or accumulation within relevant compartments, host-mediated immunomodulatory effects, or pharmacodynamic mechanisms not adequately captured by conventional *in vitro* proliferation assays. At present, however, these possibilities remain speculative and require dedicated pharmacokinetic, metabolism, and mechanistic studies before definitive conclusions can be drawn.

The present findings demonstrate that loratadine exerts a consistent and concentration-dependent inhibitory effect on *T. gondii* intracellular proliferation. The marked reduction in parasite growth at micromolar concentrations, particularly at ≥7.5 µM, indicates that loratadine interferes with essential processes required for tachyzoite replication within host cells. Importantly, the activity profile observed in the proliferation assay suggests that the compound acts more efficiently in the intracellular context, where both host- and parasite-derived factors may contribute to its antiparasitic effect.

The reversibility assay provides further insight into the nature of this activity. The sustained inhibition of parasite viability after drug removal, especially at higher concentrations, indicates that loratadine induces effects that are not reversible within the evaluated timeframe. This observation suggests a parasiticidal or long-lasting parasitostatic effect, possibly involving irreversible damage to critical parasite structures or metabolic pathways. The fact that even submaximal concentrations retained statistically significant effects after compound withdrawal reinforces the notion that loratadine may trigger persistent cellular dysfunction in the parasite. Such behavior is particularly desirable in the context of anti-*T. gondii* drug discovery, as it may reduce the likelihood of recrudescence following treatment interruption.

In contrast, the limited impact observed in assays employing pretreated extracellular tachyzoites indicates that short-term exposure to loratadine is insufficient to significantly impair parasite invasion or early intracellular establishment. Only the highest concentration tested produced a significant reduction in both invasion and subsequent proliferation, suggesting that the compound does not primarily target structures or pathways essential for host cell entry. Rather, these findings support a model in which loratadine requires prolonged exposure and/or the intracellular environment to exert its full antiparasitic effect. This may reflect the need for intracellular accumulation of the compound, metabolic activation, or interaction with host cell components that indirectly affect parasite survival.

The dose of loratadine used in this model (2 mg/kg/day) corresponds to the human-equivalent dose adjusted for body surface area [30]. The *in vivo* results are encouraging because they demonstrate that oral loratadine produced biologically meaningful outcomes in both acute and chronic models. Nevertheless, these findings should be interpreted cautiously given the exploratory nature of the animal studies. The ability to reduce brain cyst number and mean cyst area in the chronic ME49 model is particularly noteworthy because compounds capable of affecting persistent stages of *T. gondii* remain scarce. However, reductions in cyst burden and mean cyst area should not be interpreted as evidence of complete cyst eradication or direct bradyzoite killing. The present experiments cannot distinguish whether loratadine directly affects bradyzoites, interferes with cyst maintenance or maturation, or indirectly limits chronic infection through host-mediated mechanisms. Direct pharmacokinetic measurements and dedicated mechanistic studies will therefore be necessary to establish exposure–response relationships and clarify the biological basis of these observations.

Safety and translational potential are key considerations. Loratadine benefits from a long track record of clinical use as a non-sedating antihistamine, with regulatory bodies and clinical guidelines generally regarding it as acceptable for use in pregnancy when clinically indicated [31]. Nevertheless, these clinical data should not be interpreted as sufficient evidence supporting its repurposing for toxoplasmosis. Controlled human data in pregnancy remain limited, and reproductive toxicology, chronic administration studies, and efficacy evaluations under antiparasitic dosing regimens would all be required before clinical development could be considered.

Limitations of the present work include the modest *in vitro* selectivity, the absence of direct PK data linking administered dose to brain exposure, and reliance on murine models that, while informative, do not fully capture human pharmacology or the complexity of congenital toxoplasmosis. In addition, although we observed reductions in cyst number and area, it remains to be demonstrated whether loratadine can fully eradicate cysts or merely impair their growth/maintenance—a distinction with critical clinical implications.

An additional limitation is that the *in vivo* efficacy studies were conducted as exploratory experiments with relatively small group sizes and without independent biological replication. Consequently, although the observed effects were consistent across multiple outcome measures, including survival, parasite burden, and cerebral cyst parameters, these findings should be considered preliminary and interpreted with appropriate caution. Larger studies with increased statistical power and independent replication will be essential to confirm the robustness, reproducibility, and generalizability of the therapeutic effects observed here. Besides, neither tachyzoite nor tissue cyst morphology was systematically characterized in the present study. Consequently, although quantitative reductions in parasite burden and cyst size were demonstrated, the structural effects of loratadine on parasite ultrastructure, cyst integrity, or parasite differentiation remain unknown. Future studies combining histopathology, immunofluorescence microscopy, and ultrastructural analyses will be important to better understand the biological consequences of loratadine exposure on different developmental stages of *T. gondii*.

Future studies should therefore (i) measure plasma and brain concentrations of loratadine after the dosing regimen used here, (ii) perform combination studies with current anti-*Toxoplasma* drugs to assess synergism and dose-sparing potential, (iii) perform extended safety and reproductive toxicity testing if pregnancy is a target population, (iv) pursue mechanistic experiments to identify molecular targets in the parasite or host pathways involved in the observed antiparasitic activity, (v) evaluate optimized derivatives or formulations to improve selectivity and potency and (vi) investigate the pharmacokinetic and pharmacodynamic determinants underlying the apparent discrepancy between *in vitro* and *in vivo* efficacy, and (vii) evaluate larger cohorts and independent replication to further validate these findings. Collectively, these studies will be essential to determine whether loratadine itself or structurally related analogues can serve as viable starting points for anti-*T. gondii* drug development.

## 5. Conclusions

In conclusion, loratadine represents a promising lead compound for anti-*T. gondii* drug discovery, combining predicted CNS penetration, a well-characterized clinical profile, demonstrable *in vitro* antiparasitic activity, and measurable biological effects in exploratory murine models of both acute and chronic toxoplasmosis. However, its relatively low *in vitro* selectivity represents an important limitation and indicates that the present findings should be interpreted with appropriate caution. Rather than supporting immediate drug repurposing, the results provide proof-of-concept evidence that loratadine may serve as a starting point for future optimization. Additional medicinal chemistry efforts to improve potency and selectivity, together with pharmacokinetic/pharmacodynamic studies, mechanistic investigations, comparative efficacy studies including standard anti-*Toxoplasma* therapies, and larger independently replicated *in vivo* experiments, will be essential before considering clinical translation. Collectively, the present study supports further investigation of loratadine and related antihistamines as potential starting points for the development of new therapeutic strategies targeting both acute and chronic toxoplasmosis.

## Figures and Tables

**Table 1:** Pharmacokinetics and toxicity predictions of loratadine in comparison with drugs clinically used to treat toxoplasmosis.

Parameter ^1^	Loratadine	Pyrimethamine	Sulfadiazine	Spiramycin
Chemical structure	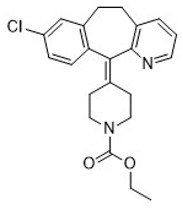	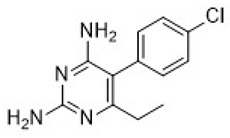	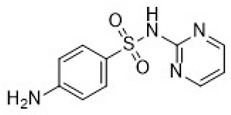	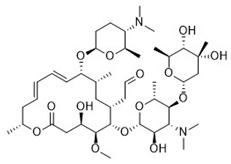
Molecular formula	C_22_H_23_ClN_2_O_2_	C_12_H_13_ClN_4_	C_10_H_10_N_4_O_2_S	C_43_H_74_N_2_O_14_
Molecular weight (g/mol)	382.88	248.71	250.28	843.05
GI Absorption	High	High	High	Low
BBB permeant	Yes	Yes	No	No
Mutagenic risk	No	Yes	No	No
Tumorigenic risk	No	Yes	No	No
Reproductive effective	No	Yes	No	No
Irritant risk	No	No	No	Yes

^1^ Chemical structure and ADME predictions obtained from http://www.swissadme.ch. Toxicity predictions were assessed using the OSIRIS software version 2.

**Table 2:** *In vitro* antiparasitic activity and cytotoxicity of loratadine and pyrimethamine.

Compound	*T. gondii* EC_50_ ± SD (μM) ^1^	HFF CC_50_ ± SD (μM) ^2^	SI ^3^
Loratadine	7.0 ± 0.9	15.5 ± 4.5	2.2
Pyrimethamine	0.2 ± 0.1	>50	>250

^1^ EC^50^: Half-maximal effective concentration against intracellular *T. gondii* tachyzoites; ^2^ CC_50_: Half-maximal cytotoxic concentration in HFF cells; ^3^ SI: Selectivity index (CC_50_/EC_50_). Data represents the mean ± standard deviation (SD) from three independent experiments.

## Data Availability

Additional information is available from the corresponding author upon reasonable request.
